# Utilization of youth friendly reproductive health services and associated factors among secondary school students in Southern Ethiopia, 2019: school-based cross-sectional study

**DOI:** 10.11604/pamj.2022.43.186.33152

**Published:** 2022-12-09

**Authors:** Destaye Teferi, Serawit Samuel, Melese Markos

**Affiliations:** 1 School of Public Health, College of Health and Medical Sciences, Haramaya University, Harar, Ethiopia,; 2 School of Public Health, College of Health Sciences and Medicine, Wolaita Sodo University, Sodo, Ethiopia,; 3 Department of Public Health, College of Health and Medical Sciences, Wachamo University, Durame, Ethiopia

**Keywords:** Youth, reproductive health, school students, Areka, Ethiopia

## Abstract

**Introduction:**

youth-friendly services are services that are accessible, acceptable, and appropriate for them. Youths are suffering by unwanted pregnancy, unsafe abortion and its complications and Sexually transmitted diseases (STIs) including HIV. However, a little study was done in Ethiopia previously. Hence, the purpose of this study is to assess the utilization of youth-friendly reproductive health services and associated factors among secondary school students in Areka Town, Sothern Ethiopia.

**Methods:**

school-based cross-sectional study design was conducted among 600 secondary school students at the Areka Town. A simple random sampling method was used to select the study participants. A structured and pre-tested self-administered questionnaire was used to collect data. Data was entered using Epi data version 3.1 and exported to the SPSS version 20 for further analysis. Adjusted odds ratio at 95% confidence interval with p-value < 0.05 was estimated to identify the associated factors on multivariable logistic regression.

**Results:**

the magnitude of youth-friendly reproductive health service utilization was 44.2%, with 95% CI: (40.0-48.4). Having sexual experience (AOR: 14.72, 95%CI (2.41-89.79)), parental monitoring (AOR = 7.65, 95% CI (1.19-49.07)) and having good knowledge (AOR: 14.02 95% CI (9.34-21.53)) were factors independently associated with youth-friendly reproductive health service utilization.

**Conclusion:**

the study shows the utilization of youth-friendly reproductive health service were low. Having sexual intercourse experience, parental monitoring, and having good knowledge were predictors of youth-friendly reproductive health service utilization. Areka Town educational office in collaboration with multimedia should provide information and create awareness on youth-friendly service.

## Introduction

The World Health Organization (WHO) classifies people in the age range of 15-24 years as youth [1]. There were 1.8 billion young people aged 10-24 years among which 1.2 billion youths aged 15-24 years globally in 2015, accounting for one out of every six people worldwide in which Africa comprises 19% (above 226 million) of the global youth population [2]. Young people make up the greatest proportion of the population in sub-Saharan Africa, with more than one-third of the population 10-24 ages [3]. Youth-friendly services are services that are accessible, acceptable, and appropriate for them.

They are in the right place, at the right price (free where necessary), and delivered in the right style to be acceptable to young people. They are effective, safe, and affordable. They meet the individual needs of young people who return when they need to and recommend these services to friends and also young people have a right to utilize youth-friendly service (YFS) [1,4,5]. Investing in youths health not only improves public health but also increases country's potential for stability, progress, and prosperity [6]. Globally STIs affect 1 in 20 young people every year, most of them are curable but many infections are left untreated. Also, HIV/AIDS affects 7000 young people every day in the world and affects disproportionally to other age groups and HIV/AIDS accounts for over 53 percent of deaths among Africa´s youth [1]. Particularly in sub-Saharan Africa including Ethiopia, youths are disproportionately affected by HIV [3]. According to the Ethiopian Demographic and Health Survey (EDHS) of 2016, among the age group of 15-24 years, HIV prevalence was the same for 15-19 and 20-24 years which is 0.2% [7]. Different studies done in different regions of Ethiopia on utilization of Youth Friendly Reproductive Health Service (YFRHS) show ranges from 21.2% to 69.1% [8-10].

Youth tend to be less informed, less experienced, and less comfortable in accessing reproductive health services than adults. Youth often lack basic reproductive health knowledge and access to affordable and confidential health services. Also, most youths do not feel comfortable discussing reproductive health issues with their parents [8]. As a result, there is a high rate of unwanted pregnancies which often result in abortions and complications. The majority (67.2%) of those seeking treatment for an incomplete abortion are under 24 years of age [1]. Existing shreds of evidence show that the major sexual and reproductive health problems of youth in Ethiopia include risky sexual practices, child marriage, early childbearing, unintended pregnancy, unsafe abortion, and its complications and STIs including HIV. Early sexual debut and teenage pregnancies are common owing to the high rate of child marriages and the subsequent family and societal pressure on girls to prove their fertility and also school dropouts are common [11].

Poor health outcomes experienced among youths were barriers to prevent young people from accessing youth-friendly reproductive health (YFRH) services include; structural barriers: such as laws and policies requiring parental or partner consent, distance from facilities, long wait times for services, inconvenient hours, lack of privacy and confidentiality, weak monitoring and evaluation, cost of services and transportation, lack of same-sex health-care providers, and socio-cultural barriers: such as restrictive norms and stigma around youth sexuality; inequitable or harmful gender norms and discrimination and judgment of youth by communities, families, peers, partners and providers and individual barriers: such as young people's limited or incorrect knowledge of sexual reproductive health, including myths and misconceptions around contraception; limited self-efficacy and individual agency were the factors associated with utilization of YFRHS [1,12].

In 2004, the Ministry of Youth, Sports, and Culture of Ethiopia developed a national youth policy to address the multi-lateral youth problems and to coordinate efforts of different stakeholders. Of the ten issues the policy tries to incorporate are youth and health, and youth and HIV/AIDS [13] even though youth are suffering from substantial negative youth-related health consequences, studies on the level of youth-friendly service (YFS) utilization and the associated factors are very limited in Ethiopia [8]. Therefore, exploring barriers to young people´s access and utilization to youth-friendly reproductive health services in Ethiopia is essential not only because of the size of their population, and also because of the need to meet the sexual reproductive health needs of young people and their roles in shaping the future of their different communities as well and the nation at large. However, few studies done in Ethiopia previously focus on single specific youth-friendly reproductive health services given under YFRHS, and also some of components of YFRH services given were missed. So this study tried to address all those service components given under the YFRH services.

## Methods

**Study area and period:** the study was conducted in Areka Town secondary schools from February 25 to March 12, 2019. The Areka Town is 184 km far from Hawassa, capital city of Southern Nations, Nationalities, and People's Region (SNNPR) state and 330 km far from Addis Ababa, capital of Ethiopia. The total population of the town is 62,252. Among those, 31,748 are females and 30,504 are males. The estimated numbers of youths are about 20,282. The town has one public youth center, 2 health cares (HCs), 7 private clinics, 5 private pharmacies, and one nongovernmental primary hospital. There are two public and one private secondary school. Totally 4,690 students were enrolled in 9 to 12 grade in all public and private schools (Areka Town Education and Health office, 2018/2019).

**Study design:** the school-based cross-sectional study design was conducted.

**Source population:** all youths attending their secondary education in Areka Town.

**Study population:** all youth students attending their education in selected secondary schools.

**Inclusion criteria:** the study included all secondary school students enrolled current year (2018/19) with ages between 15-24 years.

**Exclusion criteria:** students who were drop out at the time of data collection were excluded.

**Sample size determination:** the sample size was calculated using single population proportions at a 95% confidence level with a 5% margin of error, and the magnitude of YFRH service utilization among secondary school students in the Hadiya zone was 38.5%. The sample size was 364; by adding a 10% non-response rate and using a design effect of 1.5 due to the multistage nature of the study, n=600.

**Data collection tool:** data were collected using a structured and pre-tested self-administered questionnaire. The questionnaire was contextualized and localized to the research objectives and develops based on literature. The questionnaire was first designed in English then translated into Amharic to facilitate a better understanding of each question, and then translated back to English to check for consistency.

**Data collection facilitators and procedures:** four diploma nurses were used to facilitate data collection and two BSc. nurses were used as supervisors after providing two days of training. The supervisors provided instructions and ensured that the questionnaire is properly filled. Data were collected by using a self-administered questionnaire.

### Operational definitions

**Youth:** persons aged 15-24 years in this study.

**Service utilization:** was measured through the dichotomous response (yes or no) by asking whether a participant had utilized one or more of reproductive health (RH) service components within the last 12 months. A positive “yes” response was regarded as service utilization [9].

**Attitude:** a predisposition or a tendency to respond positively or negatively towards a certain idea, object, person, or situation. Respondents have a favorable attitude if they score equal or above the mean score of the total 8 attitude questions with 1-5 Likert scale points [9].

**Knowledge towards service:** first knowledge about the services was assessed by asking whether participants are aware of RH service components or not. Then RH knowledge was assessed through 6-item scales on service components and responding equal or above mean score was said to be knowledgeable [9].

**Data quality control:** data quality was controlled through training of data collection facilitator on objectives, questionnaire, and ways of conducting an interview. Data collection facilitators were daily supervised by 2 supervisors and report to the principal investigator on daily basis. Pre-test was done on 5% of sample size (30 students) in Bodite Town secondary school before actual data collection. After the pretest, any ambiguity, confusion, difficult words, and differences in understanding were revised based on pretest experience. The completeness and consistency of the questionnaire were checked before and immediately after the interview by each facilitator and supervisor. Simple frequencies and cross-tabulation was done for missing data, outliers, and improvable values and variables.

**Methods of data processing, analysis, and measurement:** first data was coded, completeness and consistencies of questionnaires were checked and double data entry was made using Epi data version 3.1. Then the data was exported to the SPSS version 20 for further analysis. Before analysis, data were cleaned for possible errors. Data was presented in frequency, proportions, and summary statistics to describe the study variables and factors under study. Service utilization was computed in Statistical Packages of Social Science those who respond from six RH service components at least one type in the last 12 months were considered as service utilized. It was recorded 0 as not utilized and 1 as utilized. Likert scale was used to establish the attitude of youth by scoring youths attitude using an eight-point scale that is, strongly agree =5, agree =4, undecided =3, disagree =2, and strongly disagree=1. Strongly agree and agree recoded to the same variables as agree (1) and neutral, disagree, and strongly disagree as disagree (0). Bivariate analysis was carried out to identify variables that are associated with YFRH service utilization. Multi-collinearity was diagnosed using standard error and Hosmer-Leme show test was performed to test for model fitness, variables whose p-value less than 0.25 in bivariate analysis and those fit for the model of regression were included in multivariable logistic regression. Then multiple logistic regression analysis was performed for those factors that showed an association in bivariate analysis and investigate independent predictors by controlling for possible confounders. AOR at 95% CI with p-value < 0.05 was estimated to identify the statistically significant associated factors on multivariable logistic regression.

**Ethical considerations:** the study was approved by the Institutional Health Research Ethics Review Committee of Haramaya University College of Health and Medical Sciences. The letter was written from Haramaya University to Areka Town educational office, Finally, Areka Town educational office was written to selected schools. The consent has to be informed, voluntary, written, and signed consent and ascent had attained from participants less than 18 years old youth. Data collection was made after written and signed voluntary consent and ascent is taken from each participant after informing about the study.

## Results

**Socio-demographic characteristics:** a total of 572 youths participated in the study with a 95.3% response rate, of which, 322 (56.3%) were male and 250 (43.7%) were female. Three hundred thirty-six (58.7%) falls under the age group of 15-19 and 236 (41.3%) falls under the age group of 20-24 years. The mean age was 19.2 (SD ± 2.7). Most respondents (87.4%) were single. The majority of respondents living with family 456 (79.7%) and 218 (38.1%) were in 9^th^ grade (Table 1).

**Table 1:** sociodemographic characteristics of study participants among secondary school students in Areka Town, Southern Ethiopia, 2019 (n=572)

Variable	Category	Frequencies	Percentage
Sex	Male	322	56.3
	Female	250	43.7
Age	15-19	336	58.7
	20-24	236	41.3
Residence	Urban	430	75.2
	Rural	142	24.8
Marital status	Single	500	87.4
	Others	72	12.6
Living with family	Yes	456	79.7
	No	116	20.3
Educational status	Grade 9	218	38.1
	Grade 10	174	30.4
	Grade 11	99	17.3
	Grade 12	81	14.2
Mother occupation	Housewife	315	55.1
	Merchant	172	30.1
	Civil servant	77	13.5
	Daily labor	8	1.4
Father occupation	Farmer	159	27.8
	Merchant	199	34.8
	Civil servant	197	34.4
	Daily labor	17	2.97
Mother educational status	No formal education	138	24.1
	Educated grade 1-8	247	43.2
	Educated secondary school and above	187	32.7
Father educational status	No formal education	57	10
	Educated grade 1-8	227	39.7
	Educated secondary school and above	288	50.3
Monthly income of a family	<1750	319	55.8
	1751-6500	189	33.0
	>6501	64	11.2

**Risky sexual behavior:** of all 572 study participants, 194 (33.9%) had to have a sexual partner. Of them forty-two (21.6%) had reported having multi-sexual partner, 47 (24.2%) had reported having first sexual intercourse with commercial sex workers, 153 (78.9%) had reported having sexual intercourse in the last 12 months and 49 (32.03%) of the study participants had faced RH problems in last 12 months after having sexual intercourse (Table 2).

**Table 2:** risky sexual behavior on utilization of youth-friendly reproductive health services among secondary school students in Areka Town, Southern Ethiopia, 2019

Variables	Category		Frequencies	Percentage
Having sexual partner (n=572)	Yes		194	33.6
	No		378	66.4
Number of the sexual partner (n=194)	One		152	78.12
	Multiple		42	21.6
Having first sex (N=194)	With Sexual partner/ regular		147	75.89
	With Commercial sex worker		47	24.11
Having sexual intercourse in the last 12 months (n=194)	Yes		153	78.89
	No		41	21.11
Number of sexual intercourse (n=153)	Once		29	18.95
	More than one times with the same person		92	60.13
	More than once with d/t person		32	20.91
Faced RH problem (n=153)	No		104	67.97
Faced RH problem	Yes		49	32.03
	Faced unwanted pregnancy		16	32.65
	Faced	Abortion	11	22.45
	Faced	STI	26	53.06

RH: reproductive health; STI: sexually transmitted disease

**Source of information and parental monitoring:** from all 572 study participants, near to half of the respondents, 285 (49.8%) reported that ever heard of youth-friendly reproductive health service; 62 (21.75%) heard from family, 106 (37.19%) heard from friends, 76 (26.66%) heard from teachers, 94 (49.8%) heard from newspaper and 165 (57.89%) heard from health workers as a source of information. Few, 82 (28.77%) participants discussed youth-friendly reproductive health services. Participants having parental monitoring were 149 (26%).

**Knowledge and attitude towards the service:** knowledge of respondents was assessed by using six questions and calculated their mean value, which is 3.2. Of all 572 respondents, two hundred eighty (49.0%) had good knowledge. Of the 232 (40.6%), 239 (41.8%), 88 (15.4%), 50 (8.7%), 319 (55.8%) knew family planning (FP) /condom service, voluntary counseling and testing (VCT), STI, school services and HF services respectively. Participants attitude were measured by eight attitude questions with five Likert scale, which were recorded to two scales (1=agree, 0=disagree) and using mean which is four, finally, 373 (65.2%) of the study participants were favorable attitude.

**Figure 1:**
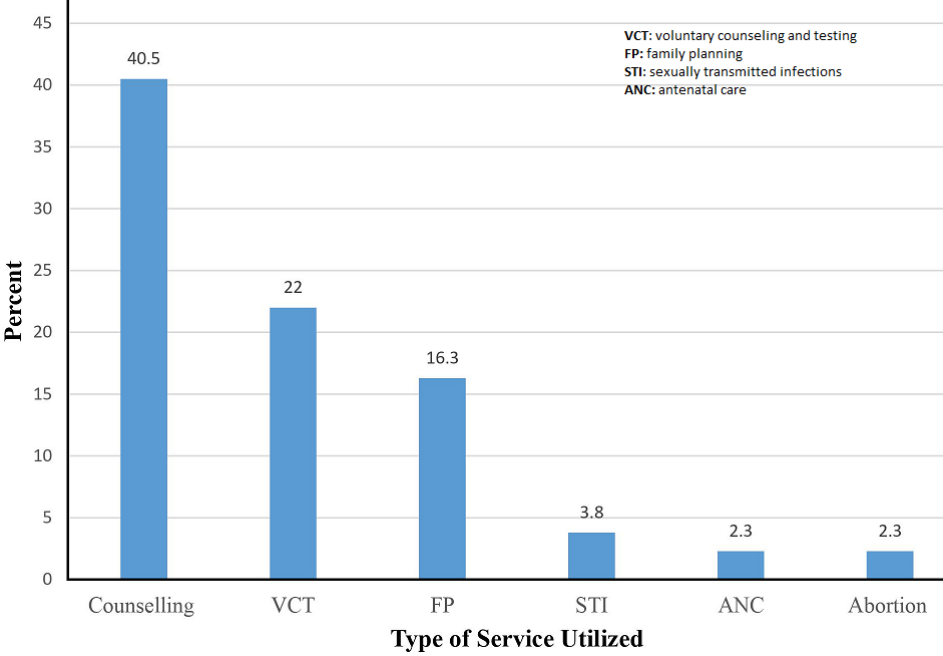
types of youth-friendly reproductive health service utilization among secondary school students in Areka Town, Southern Ethiopia, 2019 (n=572)

**The magnitude of YFRH service utilization:** the overall youth-friendly reproductive health service utilization in this study was 253 (44.2%, (95% CI: (40.0 - 48.4)). Of which, 13 (2.3%), 232 (40.5%) had utilized abortion and counseling services respectively (Figure 1).

**Places of the service utilization:** out of two hundred fifty-three (44.2%) participants utilized youth-friendly reproductive health services, one hundred seventy-five (69.17%) had utilized the service from the health center, 19 (7.5%) had utilized the service from school and seventy-two (28.45%) study participants had utilized the service from private clinics.

**Factors associated with utilization of youth-friendly RH service:** in multivariable logistic regression analyses, those sexually experienced had parental monitoring, and knowledge was significantly associated with utilization of youth-friendly reproductive health service (p<0.05). Those who had sexual intercourse in the last 12 months were 14.72 times more likely to utilize the youth-friendly reproductive health services than those who had not sexual experienced (AOR=14.72, 95% CI (2.41-89.79)), families asking their children about friends were 7.65 times utilized youth-friendly reproductive health service than those families didn't ask (AOR=7.65, 95% CI (1.19-49.07)) and having good knowledge were 14 times more likely utilized youth-friendly reproductive health services than those who had poor knowledge (AOR=14.02, 95% CI (9.34-21.53)) were significantly associated with youth-friendly RH service utilization at p-value < 0.05 (Table 3).

**Table 3:** factors associated with youth-friendly reproductive health service among secondary school students in Areka Town, Southern Ethiopia, 2019

Variables	Category	Utilization of YFRHS		COR (95%CI)	AOR (95%CI)
		Utilized	Not utilized		
Residence	Urban	207(48.1%)	223(51.9)	1.94(1.30-2.89)	0.24(0.53-15.62)
	Rural	46(32.4%)	96(67.6%)	1	1
Age group	15-19	103(30.7%)	233(69.3)	1	1
	20-24	150(63.6%)	86(36.4%)	3.95(2.77-5.61)	2.87(0.53-15.62)
Educational status	Grade 9	83(38.1%)	135(61.9)	1	1
	Grade 10	70(40.2%)	104(59.8)	1.09(0.73-1.65)	0.74(0.15-3.67)
	Grade 11	51(51.5%)	48(48.5%)	1.73(0.07-2.79)	0.38(0.06-2.66)
	Grade 12	49(60.5%)	32(39.5%)	2.49(1.48-4.20)	46.77(0.11-19952)
Marital status	Single	197(39.4%)	303(60.6)	1	1
	Others	56(77.8%)	16(22.2%)	5.38(3.00-9.65)	5.26(0.34-80.37)
Having sexual partner	Yes	154(80.2%)	38(19.8%)	0.09(0.06-0.13	0.27(0.00-1.00)
	No	99(26.1%)	281(73.9)	1	1
Living with family	Yes	180(39.5%)	276(60.5)	0.38(0.25-0.59)	0.69(0.08-5.82)
	No	73(62.9%)	43(37.1%)	1	1
Sexual experienced	Yes	133(86.9%)	20(13.1%)	5.74(2.65-12.44)	14.72(2.41-89.79) **
	No	22(53.7%)	19(46.3%)	1	1
Family monitoring	Yes	153(36.2%)	270(63.8)	3.6(2.43-5.35)	7.65(1.19-49.07) *
	No	220(77.2%)	65(22.8%)	1	1
Knowledge	Good	233(83.2%)	47(16.8%)	6.74(3.83-11.06)	14.02(9.34-21.53) ***
	Poor	20(6.8%)	272(93.2)	1	1

* P value<0.05; **P < 0.005; ***P < 0.001 (significantly associated); AOR=adjusted odds ratio; CI=confidence interval; COR=crude odds ratio; YFRHS: youth friendly reproductive health service

## Discussion

The magnitude of youth friendly reproductive health service utilization was found to be (44.2%), (95% CI 40.0 - 48.4). Moreover, sexual experience, parental monitoring, and knowledge were factors that significantly associated with the utilization of youth-friendly reproductive health services. The magnitude of youth friendly reproductive health service utilization in this study was low. This finding is lower than studies done in Kwadaso Sub-Metro of the Ashanti Region, Ghana (55.8%) [14], also in Ethiopia Harar (63%) [8]. Whereas the magnitude was greater than study done in Kenya (38.5%) [15], Nigeria (34.6) [16], Ethiopia Nekemte Town (21.2%) [9] and Bahr Dar (32%) [17]. the difference may be explained by differences in the socio-economic status, study time difference, study area, and sample size of the study.

This study revealed that sexual experience was strongly associated with youth-friendly reproductive health service utilization. Participants who had sexual intercourse in the last 12 months were 14 times more likely to utilize youth-friendly reproductive health services than those who had no sexual experience. This finding is following the study conducted at Nekemte [9] and Amhara Region [17]. This might be explained as youths who engaged in sexual intercourse were more vulnerable to reproductive health problems that might increase the need for RH services utilization. This implies that youths need access to a wide range of health information and services as well as health professional support to engage in healthy and safe behavior.

This study provides that family monitoring was significantly associated with youth-friendly reproductive health service utilization. Those families asking their children about friends were 7 times more likely to utilize the service than those families who didn´t ask. This finding is similar to the study conducted in North Shewa Amhara [10], West Hararage [18]. Participants with more parental monitoring may have better discussions on sexual issues and those who fear their parents may be more cautious to avoid any sexually-related risks. The current study revealed that knowledge towards YFS was significantly associated with YFRH service utilization. Students who had good knowledge were 14 times more likely utilized than those who had poor knowledge. This finding is following the study conducted at [8,9,18]. Participants with good knowledge of youth-friendly reproductive health services are a prerequisite to obtaining access to suitable services timely and effectively. Lastly, since this is cross-sectional study the cause and effect relationship may not be strong. So further research with strong study design like cohort is recommended.

**Limitations of the study:** since this study examines personal and sensitive issues, obtaining honest responses among youth students might be difficult. Therefore, this data might be social desirability bias. And also there may be respondent bias. Avoiding the leading question, keeping the period short and relevant, choosing the self-completion mode, and wording the question well is the way to minimize those biases.

## Conclusion

The magnitude of youth-friendly reproductive health service utilization was low. Having sexual intercourse experience, having close parental monitoring, and having good knowledge of YFRH services were predictors of youth-friendly reproductive health service utilization. Areka Town educational and health office should provide necessary materials on sexual and reproductive health services and create awareness on risky sexual behaviors.

### What is known about this topic

- Factors associated with utilization of youth friendly health service were assessed in Areka Town Secondary School, Southern Ethiopia, some of the factors identified were sexual experience, family monitoring and knowledge of respondents;
- Inadequate access to youth friendly health services were contributes to and exacerbates many of the reproductive health problems like unwanted pregnancy, HIV and sexual transmitted infections;
- Even though health services including youth friendly health services are available and acceptable, not all groups of youths can obtain the health services as they need especially in schools.

### What this study adds

- This study assesses the magnitude of utilization of youth friendly reproductive health services;
- The factors of among youth friendly reproductive health services utilization in Areka Town Secondary School students;
- Unlike age of the respondents, living with family, having sexual partner, marital status, age and educational status were did not associated with youth friendly reproductive health services utilization.
